# Bacterial Infection Elicits Heat Shock Protein 72 Release from Pleural Mesothelial Cells

**DOI:** 10.1371/journal.pone.0063873

**Published:** 2013-05-21

**Authors:** Julius F. Varano della Vergiliana, Sally M. Lansley, Jose M. Porcel, Silvia Bielsa, Jeremy S. Brown, Jenette Creaney, Suzanna E. L. Temple, Grant W. Waterer, Y. C. Gary Lee

**Affiliations:** 1 Pleural Disease Unit, Lung Institute of Western Australia, Centre for Asthma, Allergy and Respiratory Research, School of Medicine and Pharmacology, University of Western Australia, Perth, Western Australia; 2 Pleural Diseases Unit, Department of Internal Medicine, Arnau de Vilanova University Hospital, Biomedical Research Institute of Lleida, Lleida, Spain; 3 Centre for Respiratory Research and University College London, London, United Kingdom; 4 National Centre for Asbestos Related Diseases, School of Medicine and Pharmacology, University of Western Australia, Perth, Western Australia; 5 Respiratory Department, Royal Perth Hospital, Perth, Western Australia; 6 Respiratory Department, Sir Charles Gairdner Hospital, Perth, Western Australia; University of South Florida College of Medicine, United States of America

## Abstract

Heat shock protein 70 (HSP70) has been implicated in infection-related processes and has been found in body fluids during infection. This study aimed to determine whether pleural mesothelial cells release HSP70 in response to bacterial infection *in vitro* and in mouse models of serosal infection. In addition, the *in vitro* cytokine effects of the HSP70 isoform, Hsp72, on mesothelial cells were examined. Further, Hsp72 was measured in human pleural effusions and levels compared between non-infectious and infectious patients to determine the diagnostic accuracy of pleural fluid Hsp72 compared to traditional pleural fluid parameters. We showed that mesothelial release of Hsp72 was significantly raised when cells were treated with live and heat-killed *Streptococcus pneumoniae*. In mice, intraperitoneal injection of *S. pneumoniae* stimulated a 2-fold increase in Hsp72 levels in peritoneal lavage (*p*<0.01). Extracellular Hsp72 did not induce or inhibit mediator release from cultured mesothelial cells. Hsp72 levels were significantly higher in effusions of infectious origin compared to non-infectious effusions (p<0.05). The data establish that pleural mesothelial cells can release Hsp72 in response to bacterial infection and levels are raised in infectious pleural effusions. The biological role of HSP70 in pleural infection warrants exploration.

## Introduction

Bacterial pleural infection is a centuries-old disease and its global incidence continues to rise [1]. It affects 65,000 patients in the United Kingdom and United States every year [2], and carries a mortality as high as 20% [3]. *Streptococcus pneumoniae* is the leading bacterial cause of pediatric pleural infection and one of the commonest in adults [4]. Bacterial pneumonias are complicated by a simple parapneumonic effusion (PPE) in up to 40% of patients [5]–[7]. When secondarily infected, the effusion is characterised by a low fluid pH and loculation (a complicated PPE) or the presence of bacteria or frank pus (empyema) [5], [6]. The mechanism of post-pneumonic effusion development remains poorly understood [4].

Heat Shock Proteins (HSPs) are amongst the most abundant and phylogenetically conserved proteins, present in all sub-cellular compartments. HSP70 is one of the major HSPs expressed and includes the constitutive Hsp73 and stress-induced Hsp72 family members [8]. Although HSPs are traditionally thought to function as intracellular proteins, their extracellular release and activity is increasingly evident. In this regard, HSP70 can be released from both necrotic [9] and viable cells [10]–[13] in culture and Hsp72 is known to act as a potent cytokine secretagogue for various cell types, including monocytes [14], macrophages [15], fibroblast-like synoviocytes [16] and murine splenocytes [17].

The presence of extracellular HSP70 may have broad biological significance in pleural infection. HSP70 is present in various body fluids, including normal serum [18], cerebrospinal [19], [20], synovial [21] and bronchoalveolar lavage fluid [22]. Whether HSP70 is present in pleural fluid, especially in post-pneumonic effusions, has not been studied. In addition, little is known about the source of HSP70 within the pleura and the biological effects of extracellular Hsp72 on mesothelial cells have not been explored.

The present study is the first to describe the release of Hsp72 from mesothelial cells, particularly its increased release in response to *S. pneumoniae* infection *in vitro* and *in vivo,* and examine the effect of extracellular Hsp72 on mesothelial cell cytokine and chemokine release. Further, this study determined the presence of Hsp72 in human pleural fluids and describes its elevated levels in patients with infectious-related effusions.

## Materials and Methods

### Ethics statement

Animal experiments were performed on C57BL/6 and BALB/c mice aged 8–10 weeks (Animal Resources Centre, Perth, Australia). This study was carried out in strict accordance with the recommendations within the ethics application approved by the Animal Ethics Committee of the University of Western Australia (Permit number 03/100/905). All efforts were made to minimize animal suffering. Animals were anesthetized with methoxyflurane before cervical dislocation. For the use of human serum and pleural fluid samples, the Ethics Committees of Sir Charles Gairdner Hospital and Hollywood Hospital (Western Australia), the Mid- and South-Buckinghamshire and Central Oxford (UK) and the Arnau de Vilanova University Hospital (Spain) Research Ethic Committees approved the collection of the samples and all participants provided written informed consent.

### Mesothelial Cells

Primary murine mesothelial cells were harvested from the omentum of C57BL/6 mice and cultures established as previously described [23]. Primary culture of human pericardial mesothelial cells were obtained as previously described [24]. The SV40-transformed human mesothelial MeT-5A cell line was purchased from the American Tissue Culture Collection (Manassas, VA, USA). Cells were maintained in Dulbecco's Modified Eagle Medium (DMEM) supplemented with 4 mM L-glutamine, 0.2 µg/ml streptomycin, 0.2 µg/ml penicillin and fetal calf serum (FCS) at 15% (v/v) for primary cultured cells and 10% (v/v) for MeT-5A.

### Bacterial strains and culture

*S. pneumoniae* 262 (serotype 19F; ATCC #49619), TIGR4 (serotype 4; ATCC #BAA-334) and D39 (serotype 2) strains were used in this study. The 262 and TIGR4 strains were stored in Heart Brain Infusion Broth containing 15% (v/v) glycerol at −80°C and directly sub-cultured onto Blood agar plates for 18 hr at 37°C in 5% (v/v) CO_2_. Following this, suspensions were prepared in 0.85% (w/v) saline to a turbidity of 0.5 McFarland using an Oxoid Turbidometer (Thermo Scientific; Victoria, Australia). The 262 and TIGR4 strains were also subject to heat-killing at 95°C for 45 min. Viability of the live bacteria and successful heat-killing were verified by plate counts. Briefly, ten-fold dilutions of each bacteria ranging from 10^−1^ to 10^−6^ colony forming units (CFU)/ml were prepared in saline, with 20 μl spotted onto blood agar plates, and incubated overnight at 37°C. The following day, the number of CFU per 20 μl was counted and the CFU/ml calculated. The D39 strain was kindly provided by Dr Lea-Ann Kirkham (School of Paediatrics and Child Health, University of Western Australia) and were cultured on blood agar plates at 37°C over night under anaerobic conditions using the BD GasPak^TM^ EZ Anaerobic Pouch System (BD Diagnostics, Australia). Colonies were inoculated into the relevant media, grown to mid-log phase (OD_600 nm_ 0.55-0.65) and counted using a Helber bacteria counting chamber (ProSciTech; Queensland, Australia). Heat-inactivated FCS was added to the mid-log phase culture at a concentration of 20% (v/v) and stored at −80°C. Before experimentation, the D39 strain was thawed, washed and prepared to the appropriate concentration in saline.

### Immunocytochemistry

Immunocytochemistry was performed as previously described [23] using antibodies raised against Hsp72 or Hsp73 (Santa Cruz Biotechnology; Santa Cruz, CA, USA). Cells were counterstained in hematoxylin and Scott's tap water.

### Quantification of Hsp72 protein

The stressed-induced Hsp72 isoform in serum samples, pleural effusions and culture supernatants was measured using the Total HSP72/HSPA1A DuoSet IC ELISA kit, according to the manufacturer's instructions (R&D Systems; Minneapolis, MN, USA).

### Stimulation of mesothelial cells

For all experiments, cells were grown to confluence in 24-well plates and deprived of serum 24 hr prior to stimulation. For bacterial stimulations, cells were washed with antibiotic-free media and incubated with live or heat-killed *S. pneumoniae* 262 or TIGR4 (10^5^, 10^6^ and 10^7^ CFU/ml) for 2, 4, 6 and 24 hr. In separate experiments, cells were stimulated with a low-endotoxin, purified recombinant preparation of human Hsp72 (0.1–5 μg/ml) for 24 hr (Enzo Life Science; Farmingdale, NY, USA). Cells treated with 10 ng/ml phorbol 12-myristate 13-acetate (PMA; Sigma-Aldrich) were included as a positive control of cytokine release. In other studies, cells were pre-treated with Hsp72 (1 μg/ml) for 2 hr, washed and stimulated with TNF-α (5 ng/ml) (eBioscience; San Diego, CA, USA) or thrombin (1 U/ml) (Sigma-Aldrich; St Louis, MO, USA) for an additional 24 hr. Following all stimulations, the supernatants were collected and stored at −20°C until required.

### Determination of cytokine release

Monocyte chemotactic protein (MCP)-1, interleukin (IL)-10, TNF-α, interferon (IFN)-γ (eBioscience) and VEGF (R&D Systems) levels in culture supernatants were determined using ELISAs, following the manufacturer's instructions.

### Murine intraperitoneal challenge with *S. pneumoniae* D39 strain

BALB/c mice (n = 11) were given a single intraperitoneal injection of live *S. pneumoniae* D39 (∼1×10^7^ CFU in 0.1 ml of saline) or saline as a control. The viability and quantity of challenge doses were verified by parallel plate count. Mice were monitored and sacrificed by cervical dislocation at 7 and 17 hours post-injection and the peritoneal cavity was lavaged with 1 ml PBS. The lavage was centrifuged at 200×g for 10 min and the supernatant collected for quantification of Hsp72 protein (described above).

### Human pleural fluid and serum samples

Patient samples were obtained from three cohorts. Transudative and exudative effusions were defined by Light's criteria [25]. A malignant pleural effusion was defined by the presence of cancer cells on histocytologic examination of the pleural fluid or tissue biopsies. Tuberculous pleural effusion was defined by the presence of *Mycobacterium tuberculosis* on culture of pleural fluid, sputum, or pleural tissue, and/or demonstration of typical caseating granulomas on pleural biopsies. Parapneumonic effusions referred to those associated with pneumonia and were subdivided into three groups: UPPE (resolution with antibiotics alone), CPPE (non-purulent effusions which required chest tube drainage), and empyema (presence of bacteria and/or pus in the pleural cavity).

To compare the systemic and pleural fluid levels of Hsp72, blood and pleural fluid samples from 20 patients with malignant effusions and 20 with benign pleural effusions were randomly selected from the Australian Mesothelioma Tissue Bank, a member bank of the Australian Biospecimen Network-Oncology, which is supported in part by an NHMRC enabling grant [26]. The malignant effusion group included patients with malignant mesothelioma (n = 10), lung cancer (n = 4), breast cancer (n = 3) and leukemia/lymphoma (n = 3). Patients with benign effusions were tracked or followed-up until death or to last citation in the Public Health database system (Western Australia) and none had developed any pleural malignancies.

The levels of Hsp72 in different types of pleural effusions were tested in two separate cohorts. Firstly, a cohort of 273 pleural fluid samples randomly selected from a biobank of pleural fluid samples from patients undergoing diagnostic thoracenteses at the Arnau de Vilanova University Hospital (Lleida, Spain). This included 131 effusions related to bacterial infection (54 UPPE, 54 CPPE and 23 empyema), 48 tuberculous effusions, 36 malignant effusions, 18 other exudates and 40 transudates (34 congestive heart failures and 6 hepatic hydrothoraces) (Table 1).

**Table 1:** Baseline patient characteristics.

	Transudates	Malignant	Tuberculosis	UPPE	CPPE	Empyema	Miscellaneous exudates	p value
**Spain cohort:**								
Subjects, n	40	36	48	54	54	23	18	
Age, yr	82 (73–87)^+^	75 (66–82)	35 (28–44)*	54 (40–71)	62 (43–80)	70 (51–75)	70 (59–61)	<0.001
Male sex, n	23 (58)	17 (47)	33 (69)	30 (56)	38 (70)	15 (65)	11 (61)	0.308
Hsp72, ng/mL	7 (4–13)*	13 (5–26)*	21 (10–38)	27 (11–161)	23 (12–72)	18 (10–356)	17 (11–23)	<0.001

Data are presented as median (quartile range) or n (%).

+Significantly higher than the respective values in other groups by *post-hoc* test.

* Significantly lower than the respective values in other groups by *post-hoc* test.

The results were further verified in a validation cohort comprising pleural fluid prospectively collected from patients (n = 243) presenting with pleural effusions for diagnosis/management at the Oxford Pleural Unit (Oxford, UK), as reported previously [27]. In brief, this cohort included 243 patients, including 36 effusions related to bacterial infections (9 UPPE, 1 CPPE and 26 empyema), 3 tuberculosis effusions, 151 malignant pleural effusions, 37 others exudates and 16 transudates (12 congestive heart failures and 4 hepatic hydrothoraces).

### Statistical analysis

Data are presented as mean ± standard error of the mean (SEM and median (range), where appropriate. A *p* value of <0.05 was considered statistically significant.

The differences among groups were compared by one-way analysis of variance (ANOVA) on-ranks with multiple comparisons between groups performed using Dunn's post hoc test. Student's *t* test with Bonferroni correction and Wilcoxon Rank Sum Test were used to compare differences between two treatment groups for parametric and non-parametric data, respectively. Paired *t* test was used to compare differences between matched serum and pleural fluid samples. The discriminative properties of Hsp72 were evaluated using receiver operating characteristics (ROC) curve analysis, selecting cut-off values with more than 80% specificity for infectious effusions. Measures of diagnostic accuracy (sensitivity, specificity, likelihood ratios (LR)) of pleural fluid Hsp72 for differentiating effusions of infective causes (PPE and empyema) from those of non-infective etiologies, PPE *vs* non-PPE, and CPPE *vs* UPPE, were calculated. Analyses were conducted using statistical softwares: SPSS v18.0 (Chicago, IL, USA) and GraphPad Prism 4.0 (La Jolla, CA, USA).

## Results

### Mesothelial cells express HSP70

**Figure 1:**
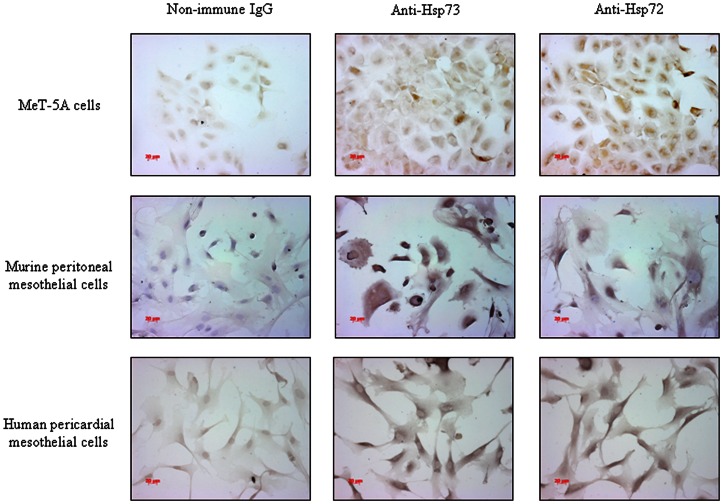
Mesothelial cells express Hsp72 and Hsp73 on the cell surface. Non-permeabilised mesothelial cells were assessed for expression of cell surface-associated HSP70 proteins by immunocytochemistry. The constitutive Hsp73 and stress-induced Hsp72 forms were examined separately. The figures are representative of three independent experiments. Bar  = 20 μm.

Using non-permeabilised mesothelial cells, we demonstrated for the first time that mesothelial cells are a source of both the constitutive Hsp73 and stress-induced Hsp72 isoforms of HSP70. All human and murine mesothelial cells tested expressed Hsp73 and Hsp72, as judged by immunocytochemistry. The total cell population of each mesothelial cell type stained for Hsp73 and Hsp72. For MeT-5A cells, Hsp72 immunoreactivity was greater compared to Hsp73, whereas Hsp73 staining was more intense for the primary mesothelial cell types tested (Figure 1).

### Live and heat-killed *S. pneumoniae* induce Hsp72 release from mesothelial cells *in vitro*

**Figure 2:**
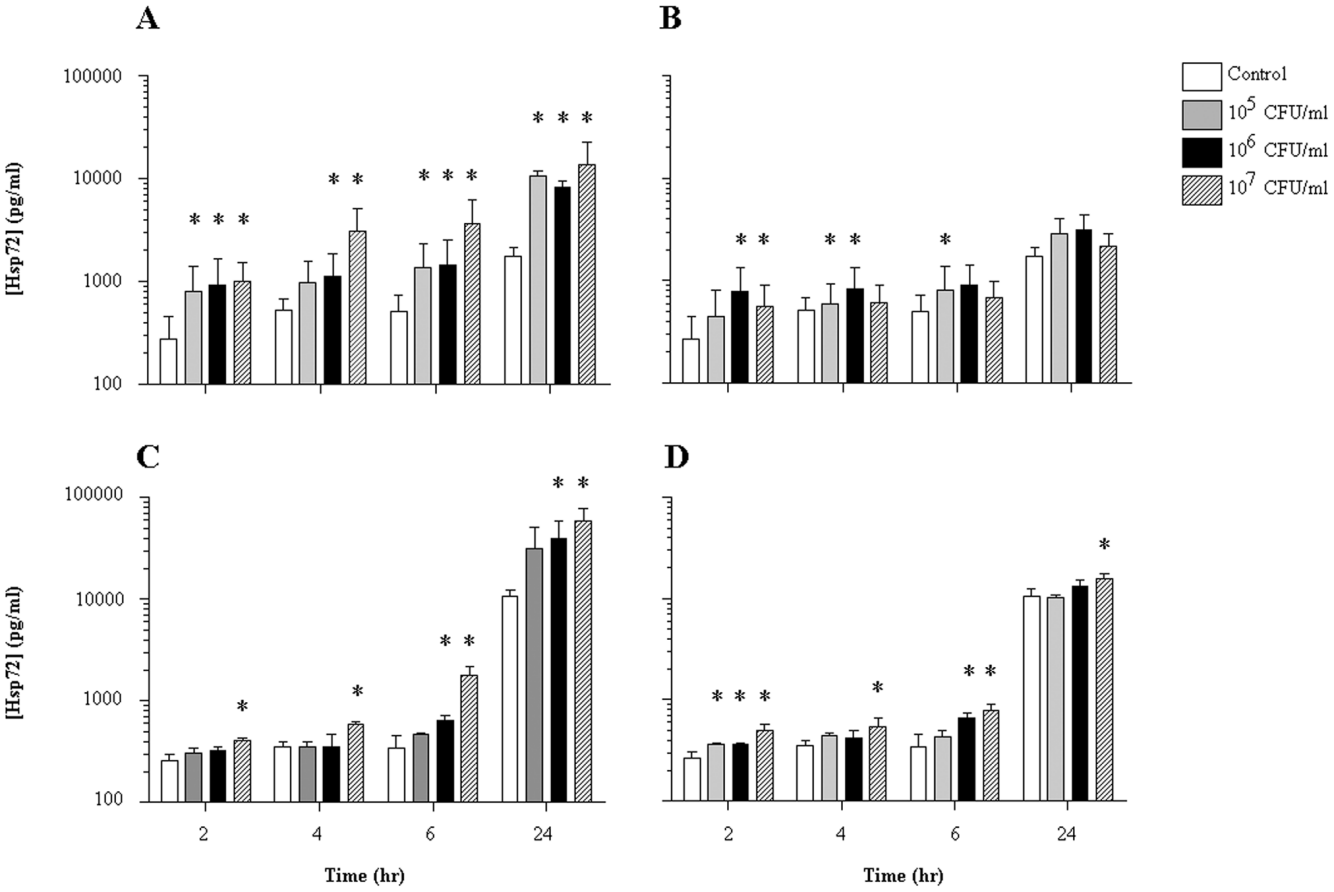
Pleural mesothelial cells release Hsp72 in response to infection with *Streptococcus pneumoniae*. MeT-5A cells were treated with live (A and C) or heat-killed (B and D) *Streptococcus pneumoniae* 262 strain (A and B) and *S. pneumoniae* TIGR4 strain (C and D) and Hsp72 levels measured in culture supernatants at various time points up to 24 hr by ELISA. Significant release of Hsp72 was observed at all time points examined (p<0.05) and was still evident following treatment with heat-killed *S. pneumoniae.* * Denotes significantly higher than vehicle control cells (p<0.05). The data are presented as the mean ± SEM of three independent experiments performed in triplicate.

MeT-5A cells were treated with live or heat-killed *S. pneumoniae* strains and released Hsp72 measured in culture supernatants by ELISA. Hsp72 was released from cultured MeT5A mesothelial cells at all time points examined. Live *S. pneumoniae* 262 and TIGR4 both induced Hsp72 release from mesothelial cells (p<0.05) (Figures 2a and 2c). The stimulation of Hsp72 release was still observed for both *S. pneumoniae* strains even when the bacteria were heat-killed before experimentation, but, to a lesser extent (Figures 2b and 2d).

### Hsp72 does not induce selected cytokine release from pleural mesothelial cells

**Figure 3:**
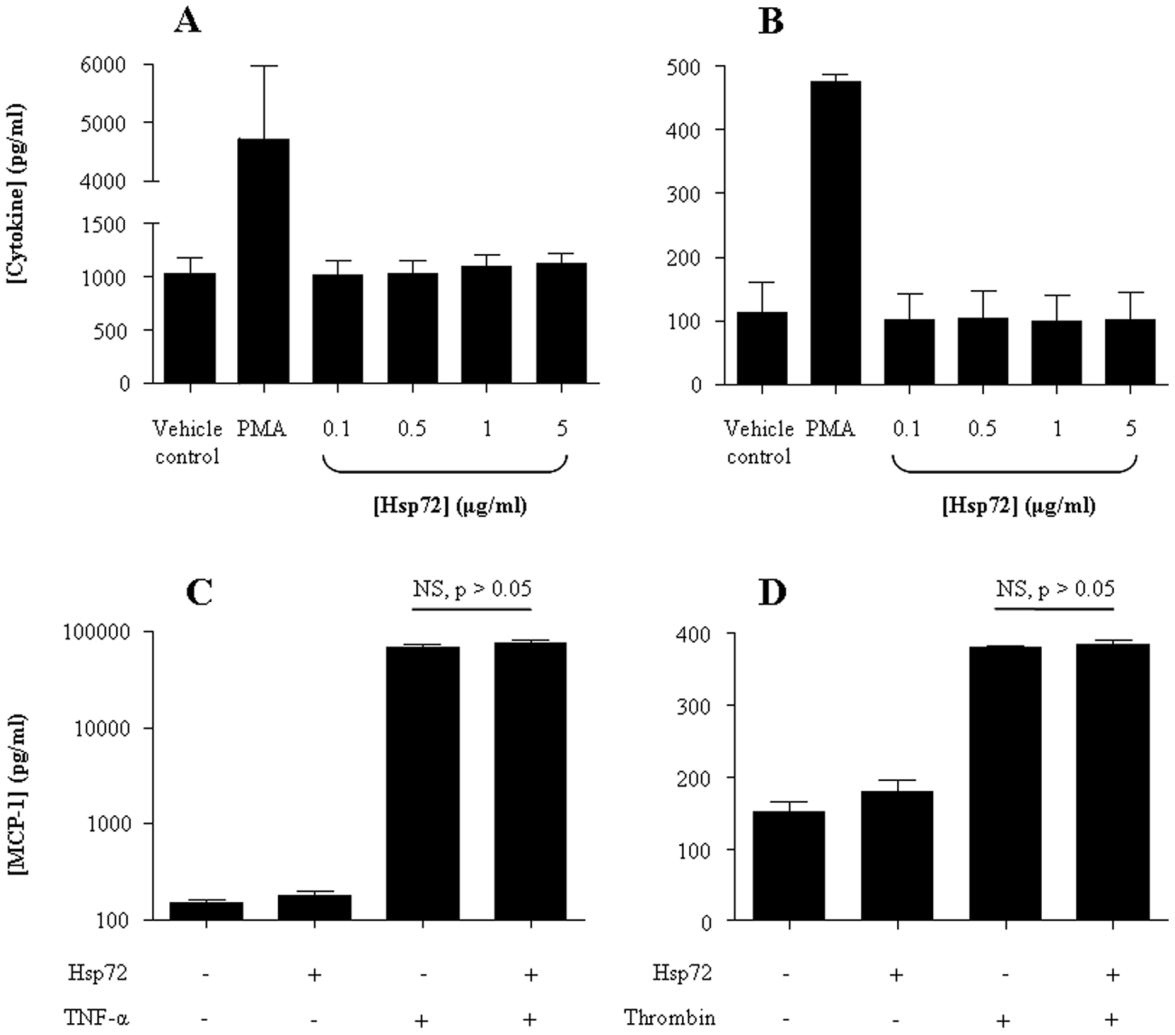
Extracellular Hsp72 does not induce or inhibit cytokine release from pleural mesothelial cells. The ability of a highly purified, recombinant Hsp72 preparation to induce inflammatory cytokine release from pleural mesothelial cells was determined (A and B). MeT-5ACells were treated with the indicated doses of Hsp72 or PMA as a positive control, and MCP-1 (A) and VEGF (B) release measured 24 hr post-treatment by ELISA. In separate experiments, the anti-inflammatory effects of extracellular Hsp72 were examined (C and D). Cells were pre-treated with Hsp72 for 2 hr, followed by treatment with TNF-α (C) or thrombin (D) for an additional 24 hr to induce MCP-1 release. In contrast to PMA stimulated MeT-5A cells, treatment with Hsp72 had no impact on cytokine release. The data are presented as the mean ± SEM of three independent experiments performed in triplicate. NS, not significant.

The stress-induced Hsp72 isoform can induce cytokine release in some cell types [14], and paradoxically inhibit cytokine-induced mediator release from others [28]. Its ability to do so in mesothelial cells has not been tested. MeT-5A cells were, therefore, stimulated with highly purified recombinant Hsp72 and the supernatant was measured for cytokine release. In contrast to PMA treatment, Hsp72 stimulation had no effect on mesothelial cell release of MCP-1 and VEGF (Figure 3a and 3b) or TNF-α, IL-10 and IFN-γ (data not shown). Pre-treatment of MeT-5A cells with Hsp72 did not change TNF-α- or thrombin-induced MCP-1 levels released from MeT-5A cells (Figures 3c and 3d).

### *S. pneumoniae* induced Hsp72 release *in vivo*

**Figure 4:**
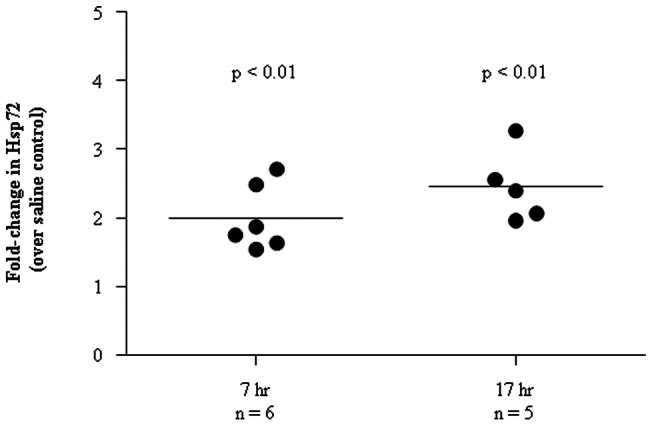
Hsp72 levels in peritoneal lavage are elevated following intraperitoneal injection of mice with *Streptococcus pneumoniae*. BALB/c mice were given a single intraperitoneal injection of live *Streptococcus pneumoniae* D39 strain (∼1×107 CFU in 0.1 ml of saline; n = 11) or saline as a control (n = 10). The peritoneal cavity was lavaged 7 (n = 6) and 17 hr (n = 5) post-injection with 1 ml PBS for quantification of Hsp72 protein. The results are presented as the fold-increase in Hsp72 levels over the mean Hsp72 level in mice injected with saline alone. An approximately 2- and 2.5-fold increase in Hsp72 was shown peritoneal lavage following infection with *S. pneumoniae* for 7 and 17 hr, respectively (p<0.01 for both).

To further confirm that bacterial infection contributes to Hsp72 release in serosal cavities lined by mesothelial cells, mice were injected intraperitoneally with live *S. pneumoniae* D39. A 2-fold increase in Hsp72 concentrations were found in the peritoneal lavage of mice treated with *S. pneumoniae* D39 over saline controls at 7 hr post-injection (p = 0.0087). A similar increase in Hsp72 levels was observed at 17 hr post-injection, with the peritoneal lavage Hsp72 levels being ∼2.5-fold higher in mice injected with D39 (p = 0.0079) (Figure 4).

### Hsp72 levels in patients with bacterial pleural infection

To further confirm that Hsp72 was released by pleural mesothelial cells in response to bacterial pleural infection three patient cohorts were used. First, systemic and pleural Hsp72 levels were compared in an Australian cohort. Second, in a Spanish cohort Hsp72 levels in infectious and non-infectious patients were determined and the diagnostic accuracy of pleural fluid Hsp72 compared to traditional pleural fluid parameters. Third, these results were further verified using a UK cohort.

**Figure 5:**
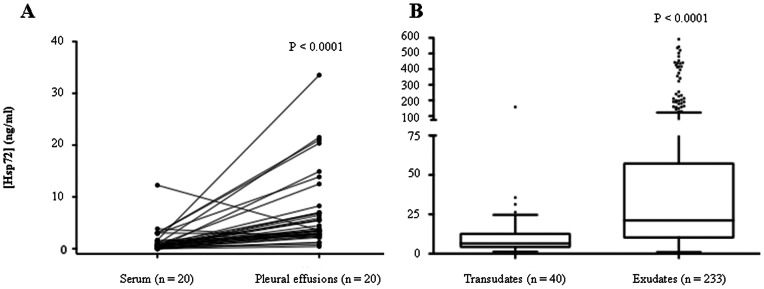
Hsp72 levels are elevated in exudative pleural effusions. Hsp72 was measured in human samples by ELISA and levels compared between pleural fluid and serum (A), and transudates and exudates (B). A) Median Hsp72 levels were significantly elevated in pleural fluid compared to their matched serum sample (n = 20 for each group) (3.6 vs. 0.49 ng/ml; p<0.0001). B) Median Hsp72 levels were higher in exudates (n = 233) compared to transudates (n = 40) (21.2 *vs* 6.5 ng/ml, p<0.0001).

Hsp72 was detected in all human pleural fluids obtained from patients in all three cohorts (combined n = 536). In contrast, Hsp72 was detectable in the sera of only 15 of the 20 patients tested. The median Hsp72 concentration was 7.25 fold higher in pleural fluid compared to serum (3.6 vs. 0.49 ng/ml; p<0.0001). Pleural fluid Hsp72 concentration was higher (by up to 300 fold) than the corresponding serum in the majority (92.5%) of patients tested (Figure 5a). These findings were consistent with a cellular source of Hsp72 from within the pleural cavity.

**Figure 6:**
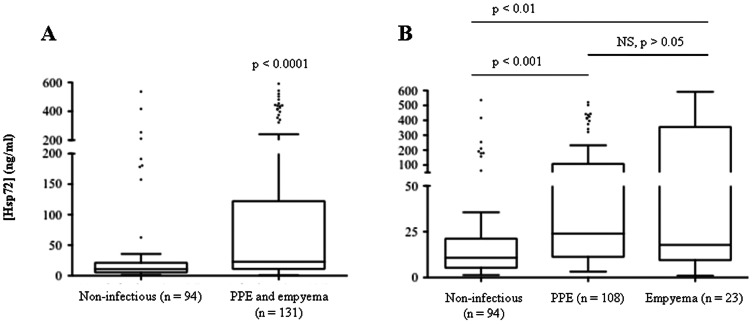
Pleural fluid Hsp72 levels in infection-related and non-infective effusions. Pleural fluids Hsp72 levels were compared among infection-related and non-infective effusions in the Spanish cohort. A) Median Hsp72 levels were significantly higher in infection-related pleural effusions compared to effusions of non-infective etiologies (p<0.0001). B) Compared to non-infective effusions, Hsp72 levels were also higher when in PPE (p<0.001) and empyema (p<0.01).

Pleural fluids Hsp72 levels were compared among patients with infective and non-infective effusions in the Spanish cohort. The median pleural fluid Hsp72 concentrations were higher in exudates compared to transudates (21.2 *vs* 6.5 ng/ml, p<0.0001) (Figure 5b). Pleural fluid Hsp72 levels were significantly higher in patients with infection-related pleural effusions (PPE and empyema) over those with non-infective etiologies (23 *vs* 10.8 ng/ml, p<0.0001) (Figure 6a). This finding remained robust if tuberculous effusions were included in the infectious group over the non-infectious group (22.9 *vs* 10.8 ng/ml, p<0.0001). When analysed separately, the PPE and empyema groups had significantly elevated Hsp72 levels compared to the non-infective effusion group (p<0.001 and p<0.01, respectively). However, no significant difference in pleural fluid Hsp72 was observed between PPE and empyema patients (Figure 6b). Similar results were obtained when pleural fluid Hsp72 levels were measured in the UK cohort. The median pleural fluid Hsp72 levels were higher in exudates compared to transduates by approximately 1.8-fold (p = 0.041). Pleural fluid Hsp72 levels were elevated in patients with infection-related effusions (PPE and empyema) compared to non-infective etiologies by 1.5-fold (p = 0.02) and were the highest in patients with empyema (p<0.05).

The value of pleural fluid Hsp72 to discriminate between infection-related effusions and those of non-infective etiologies was examined in the Spanish cohort. Using a threshold of 40 ng/ml, pleural fluid Hsp72 could help separate infection-related effusions from non-infective ones or PPE from other etiologies, although the AUC was modest (0.688 and 0.658 respectively) and was inferior to conventional biomarkers such as pH, glucose and LDH used for the differentiation of UPPE and CPPE (Table 2).

**Table 2:** Measures of diagnostic accuracy of pleural fluid Hsp72.

Pleural fluid Hsp72, ng/mL	Sensitivity, %	Specificity, %	LR+	LR-	AUC
**Infectious vs. Non-infectious**					
≥15	62 (55–69)	62 (52–71)	1.6 (1.2–2.1)	0.62 (0.48–0.79)	0.688 (0.624–0.753)
≥20	57 (50–64)	72 (63–80)	2.1 (1.5–2.9)	0.60 (0.48–0.73)	
≥25	49 (41–56)	82 (73–88)	2.7 (1.7–4.2)	0.63 (0.53–0.74)	
≥40	31 (25–38)	90 (83–95)	3.3 (1.7–6.3)	0.76 (0.68–0.86)	
≥50	28 (22–36)	90 (83–95)	3.0 (1.5–5.8)	0.79 (0.71–0.87)	
**PPE vs. other etiologies**					
≥25	49 (40–57)	72 (64–79)	1.7 (1.3–2.4)	0.71 (0.59–0.87)	0.658 (0.593–0.723)
≥30	45 (37–54)	80 (72–85)	2.2 (1.5–3.2)	0.69 (0.58–0.82)	
≥40	34 (27–43)	86 (79–91)	2.4 (1.5–3.9)	0.76 (0.66–0.88)	
≥50	33 (25–41)	88 (82–92)	2.7 (1.6–4.6)	0.76 (0.67–0.87)	
**CPPE vs. UPPE**					
pH ≤7.20	63 (50–75)	87 (74–93)	4.7 (2.3–9.7)	0.42 (0.29–0.61)	0.831 (0.75–0.912)
Glucose ≤60 mg/dL	63 (50–75)	93 (82–97)	8.5 (3.2–22.3)	0.4 (0.28–0.57)	0.826 (0.742–0.909)
LDH ≥1500 UI/L*	57 (44–70)	81 (69–90)	3.1 (1.7–5.7)	0.52 (0.37–0.73)	0.753 (0.662–0.845)
Hsp72 ≥40 ng/mL	37 (24–51)	69 (54–80)	0.9 (0.5–1.4)	1.1 (0.8–1.4)	0.55 (0.438–0.66)

Values in parentheses are 95% confidence intervals.

LR, likelihood ratio; AUC, area under ROC curve.

* This figure represents three times the upper normal limit for serum LDH.

## Discussion

Although previous reports have demonstrated inducible changes in HSP70 expression in peritoneal mesothelial cells [29], this is the first study to show its extracellular release from pleural mesothelial cells upon bacterial stimulation *in vitro* and *in vivo*. The findings were further confirmed in three cohorts of human pleural fluid samples, which showed significantly elevated Hsp72 levels in infection-related effusions. Although our data suggests the extracellular release of Hsp72 contributes to the elevated pleural fluid levels in infection-related effusions, it is possible that impaired clearance of Hsp72 from the pleural space can contribute to its elevated levels in pleural fluid. Our data provide the platform for future investigations of the role of Hsp72 in the pathophysiology of bacterial pleural infection.

Pleural infection remains a significant global health problem with a rising incidence in reports from most regions around the world [1]. The pathobiology of pleural infection however remains poorly understood. Mesothelial cells line the pleural, peritoneal and pericardial cavities and are the most abundant cell type in all these serosal cavities. Mesothelial cells are biologically active and express a broad range of inflammatory mediators, especially in response to undesirable infiltrating molecules including microbes [30].

HSP70 is present in all organisms and, in unstressed conditions, maintains intracellular protein homeostasis by mediating the folding and translocation of naïve proteins. Although produced constitutively, HSP70 is abundantly expressed in response to various physiological, environmental and pathological stressors [8]. Bacterial infection is known to be a potent inducer of HSP70 expression in variety of cell types [31], [32]. Previous reports have associated infectious diseases with elevated HSP70 levels in body fluids, including in plasma of children during septic shock [33], in cerebrospinal fluid [19] and in seminal plasma of the prostate [34]. However, the presence and biological relevance of HSP70 in pleural infection and the cellular source of pleural fluid HSP70 have not been clarified. This study demonstrates that pleural mesothelial cells are a source of extracellular HSP70 in pleural infection. Why MeT-5A cells demonstrated differential staining of Hsp72 and Hsp73 is unclear and should be the focus of further research.

We demonstrated that mesothelial cells produce Hsp72 through three lines of experiments: in cell culture, in a murine model and in three human pleural effusion cohorts totaling over 500 patients. The finding is therefore robust, and is likely to be representative of peritoneal and pericardial infections. In nearly all patients studied, pleural fluid Hsp72 concentrations were higher than the corresponding sera, suggesting Hsp72 was largely produced within the pleural space. As such, our study is the first to demonstrate the extracellular expression and release of Hsp72 by pleural mesothelial cells. Although mesothelial cells are a source of extracellular Hsp72 within the pleural cavity, Hsp72 release from other cell types such as neutrophils [35] and monocytes [36] likely contribute to the overall HSP70 pool within pleural effusions.

Hsp72 release was upregulated from mesothelial cells in response to *S. pneumoniae*, whether live or heat-killed. This finding suggests that bacterial stimulation of Hsp72 release is mediated at least in part via structural molecules within the bacteria and not predominantly dependent on secreted bacterial products. It is noteworthy that prior studies have shown HSP70 release by necrotic, but not apoptotic, cells [9]. Therefore, the liberation of intracellular Hsp72 following cell lysis as a result of bacterial exposure may contribute to the elevated pleural fluid Hsp72 concentrations observed from patients with empyema in whom mesothelial cell necrosis are common. When cocultured, *S. pneumoniae* can induce death of mesothelial cells within 24 hr (data not shown), a process that can contribute to the increased levels of extracellular Hsp72 observed. However, increased Hsp72 release is found when cells were treated with heat-killed bacteria (which induce minimal cell death) as well as at earlier time points of infection with live bacteria when no cell death was observed. We are therefore confident that viable mesothelial cells release Hsp72 in response to bacteria. The precise interactions between bacteria and mesothelial cells that trigger Hsp72 release was beyond the scope of this study.

The purpose of the mesothelial release of Hsp72 upon bacterial infection is unclear. Hsp72 is often described in the past as a chaperone protein. However, recently extracellular functions have been attributed to Hsp72 and it is now described as a “chaperokine” to reflect its dual role as a chaperone and cytokine. Hsp72 is known to elicit intracellular calcium mobilisation [14], induce NF-κB activity [17] and, most notably, stimulate the release of pro-inflammatory cytokines [14], [37]. We used a highly purified preparation of Hsp72, but failed to induce cytokine and chemokine release from mesothelial cells. This may be explained by recent findings that the *in vitro* cytokine effects of Hsp72 are due to molecules bound to Hsp72 or contaminants present in the preparations used, namely bacterial endotoxin [38]–[41]. It is also possible that higher concentrations of Hsp72 are required to induce pro-inflammatory cytokine release from mesothelial cells, as seen in macrophages [42]. Further, Hsp72 may act on other (e.g. inflammatory) molecules and cells within the pleura directly, or via paracrine pathways, which could coordinate/modulate pleural inflammation via cytokine crosstalk in the pleural cavity [43]. In addition, the constitutive Hsp73 isoform has also been shown to have cytokine effects *in vitro* [44] and, therefore, may induce similar responses in mesothelial cells.

*In vitro*, extracellular Hsp72 has also been shown to act as an anti-inflammatory mediator inhibiting pro-inflammatory cytokine release [28]. Further, an anti-inflammatory role has been proposed *in vivo* using HSP70 deficient mice [45]–[47]. Inducible changes in Hsp72 have been shown to contribute to the resolution of inflammation in a rat model of carrageenin-induced pleurisy [48], [49]. Thus, contrary to its proposed pro-inflammatory activity, HSP70 may play an anti-inflammatory role in pleural infection. *In vitro* we were unable to demonstrate effect of extracellular Hsp72 on pro-inflammatory cytokine release. The precise biologic effect of released HSP70 proteins in pleural infection warrants further investigation.

A chaperone protein may be useful clinically as a biomarker for pleural disease. In this regard, HSP70 expression of effusion cytology has been shown to be a prognostic marker of poor survival in malignant disease [50]. However, our study is the first, to our knowledge, to assess extracellular HSP70 levels in pleural fluid. The pleural fluid level of Hsp72 was indeed raised in infection-related effusions, and showed a graded increase from simple to CPPE to frank empyema. The discriminatory value of Hsp72 was, however, inferior to conventional markers (eg pleural fluid pH). This may be due to a variety of clinical reasons including that confounding interactions among these markers (all of which reflects pleural inflammation), timing of collection of fluid and other patient comorbidity that may influence the Hsp72 levels. Nonetheless, the human fluid Hsp72 data further support that HSP70 forms part of the mesothelial response to bacterial invasion of the pleura, and the potent and consistent release of Hsp72 may have a biologic role rather than acting purely as a chaperone.

In summary, this study confirms the ‘sufficiency’ of the mesothelium as a pleural source of extracellular Hsp72 and provides a rationale for the elevated Hsp72 levels observed in infectious pleural effusions. We demonstrate for the first time the extracellular release of Hsp72 in response to live and heat-killed bacteria from mesothelial cells. Further studies are required to elaborate on the extracellular functions of HSP70 in pleural infection.
